# Fibrosis Process Activation in Patients with Acute Cardiac Rejection: A Novel Noninvasive Diagnostic Approach

**DOI:** 10.3390/biomedicines14061371

**Published:** 2026-06-18

**Authors:** Marta Delgado-Arija, Lorena Pérez-Carrillo, Irene González-Torrent, Patricia Genovés, Isaac Giménez-Escamilla, Carlota Benedicto, Luis Martínez-Dolz, Manuel Portolés, Estefanía Tarazón, Esther Roselló-Lletí

**Affiliations:** 1 Clinical and Translational Research in Cardiology Unit, Health Research Institute Hospital La Fe (IIS La Fe), Avd. Fernando Abril Martorell 106, 46026 Valencia, Spain; marta_delgado@externos.iislafe.es (M.D.-A.); lorena_perezc@iislafe.es (L.P.-C.); irene_gonzalez@iislafe.es (I.G.-T.); isaacgimenezuv@gmail.com (I.G.-E.); carlota_benedicto@iislafe.es (C.B.); martinez_luidol@gva.es (L.M.-D.); drmanuelportoles@gmail.com (M.P.); 2 Center for Biomedical Research Network on Cardiovascular Diseases (CIBERCV), Avd. Monforte de Lemos 3-5, 28029 Madrid, Spain; pagenomar@gmail.com; 3 Department of Biomedical Sciences, CEU Cardenal Herrera University, Street Santiago Ramón y Cajal 20, 46115 Valencia, Spain; 4 Heart Failure and Transplantation Unit, Cardiology Department, University and Polytechnic La Fe Hospital, Avd. Fernando Abril Martorell 106, 46026 Valencia, Spain

**Keywords:** fibrosis, acute cellular rejection, heart transplantation

## Abstract

**Background/Objectives**: Cardiac allograft fibrosis is an important limiting factor for long-term graft survival. However, the fibrotic process operating in patients with acute cellular rejection (ACR) remains unclear. We aimed to identify altered serum mRNAs related to cardiac fibrosis in patients with ACR and to evaluate their diagnostic accuracy in detecting rejection episodes. **Methods**: We included 40 serum samples from recipients of transplants undergoing routine endomyocardial biopsies. **Results**: Several altered mRNAs associated with fibrosis were detected in patients with ACR. Specifically, the activators of fibroblasts and myofibroblasts (*TNS1*, *FAP* and *ACTA2*), TGF-β signaling (*TGFBR1* and *JAK1*) and WNT signaling (*WNT7A* and *WLS*) pathways were significantly different when we compared grade ≥ 2R ACR and/or grade 1R ACR groups with the nonrejection group. Furthermore, *TNS1* and *WLS* presented an area under the curve value > 0.90 for identifying patients with moderate and severe grades of cardiac rejection. **Conclusions**: In conclusion, we found alterations in the relative abundance of circulating activators of fibroblasts and myofibroblasts, such as *FAP* or *ACTA2*, as well as in major profibrotic pathways, including TGF-β and WNT signaling, especially in clinically relevant cardiac rejection. These findings may contribute to improving the surveillance of patients with cardiac transplant and provide new therapeutic strategies for targeting fibrosis process activation.

## 1. Introduction

Heart transplantation remains the most effective therapeutic option for patients with end-stage heart failure (HF) [1]. Despite major advances in immunosuppression, allograft rejection remains a significant problem in the first year following heart transplantation, leading to adverse short- and long-term outcomes [2]. The gold standard method for monitoring and diagnosing cardiac allograft rejection continues to be the evaluation of pathologic cardiac tissue obtained through an endomyocardial biopsy (EMB) [1], although this procedure has potential negative consequences for patients [3] and is subject to sampling error and interobserver variability [4]. Therefore, noninvasive methods and reliable biomarkers are being actively sought to screen for heart transplant rejection and to better understand the pathophysiology of this post-transplant complication [5].

The pathophysiological process involved in cardiac allograft rejection is complex and is characterized by severe cardiac inflammation, cardiomyocyte damage, and progressive ventricular dysfunction in the transplanted heart [6]. In addition to inflammation, cardiac allograft fibrosis has been identified as an important limiting factor for long-term graft survival [6,7]. Fibrosis is one of the major causes of cardiac allograft malfunction and is mainly driven by fibroblasts [8]. Myocardial fibrosis is a described histopathological feature in recipients of heart transplants, and several studies have demonstrated the appearance of this condition during time course analyses in different populations [9,10,11,12]. Whether acute cellular rejection (ACR) in recipients of heart transplants is associated with myocardial fibrosis remains unclear.

Organ fibrosis is characterized by the excessive deposition of extracellular matrix (ECM) proteins within the tissue and around blood vessels [13]. Several effectors and molecular mechanisms are involved in fibrosis. Among them, activated fibroblasts are central cellular effectors that serve as the main ECM-producing cells [14]. Activated fibroblasts and myofibroblasts are well recognized as significant contributors to graft fibrosis [15]. In addition, transforming growth factor beta (TGF-β) is the best characterized fibrogenic growth factor, and its activation has been consistently demonstrated in different models of cardiac fibrosis and in human hearts with fibrotic cardiomyopathic changes [14]. Another pathway involved in fibrosis is WNT signaling, which is activated in many cell types and contributes to reparative, regenerative, and fibrotic responses [14]. Interestingly, it has been proposed that the crosstalk between TGF-β and Wnt/β-catenin pathways regulates cardiac fibrosis [16].

However, the relationship between this process and cardiac allograft rejection remains unknown. For this reason, we used serum samples of patients diagnosed with ACR to analyze mRNA relative abundance of the genes implicated in the fibrotic process, including ECM components, such as collagens, and activators of fibroblasts and myofibroblasts. Furthermore, we examined the relative abundance of genes encoding for proteins involved in the major pathways of the fibrotic process, such as TGF-β and WNT signaling. Finally, we assessed the diagnostic capability of those altered molecules related to the fibrosis process in patients with ACR.

## 2. Materials and Methods

### 2.1. Sample Collection

**Figure 1:**
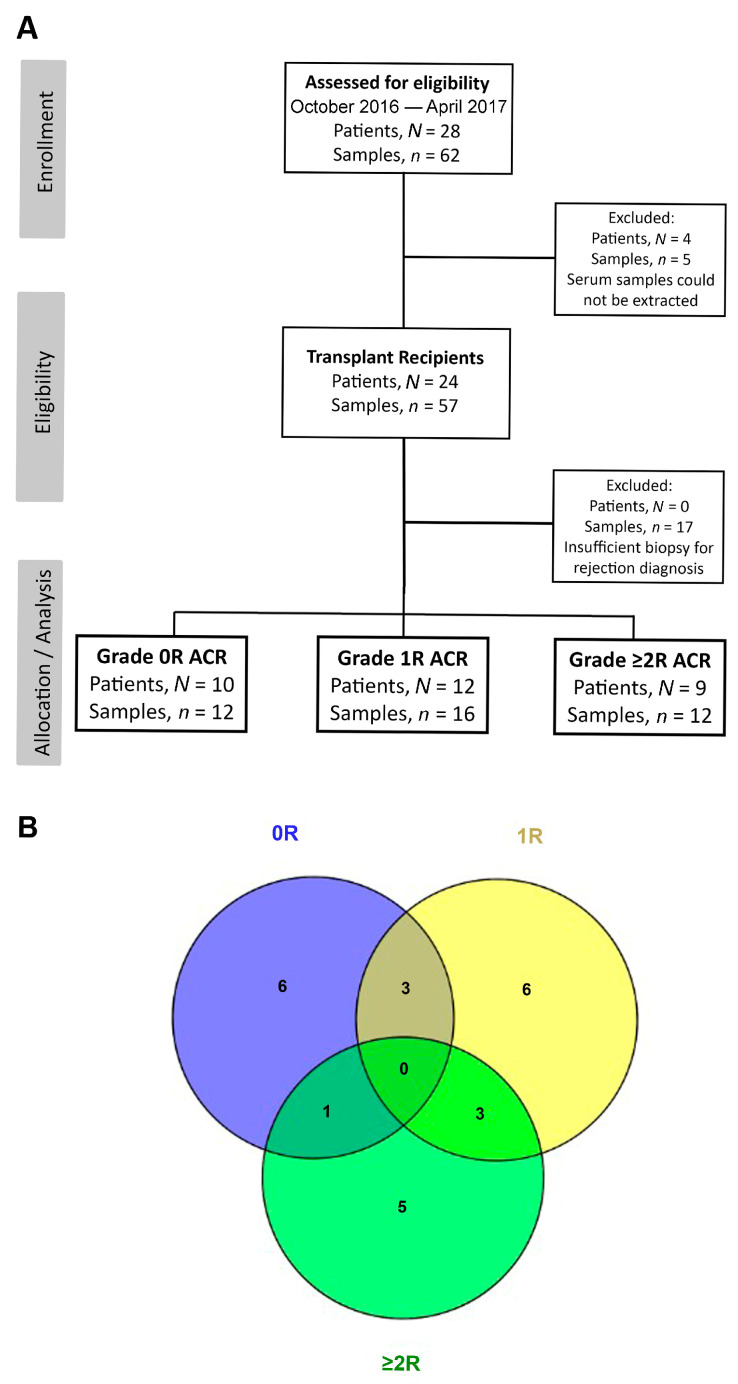
Samples and patients included in this study. CONSORT flow diagram showing patient and sample enrollment, eligibility and exclusions, as well as the allocation depending on the degree of cardiac rejection for the final analysis (**A**). Venn diagram showing patients with analyzed samples belonging to more than one study group (**B**). ACR, acute cellular rejection.

This study included serum samples collected from patients (>18 years old) who received a heart transplant and were referred for EMB as a scheduled routine screening at a single center. All consecutive patients who underwent primary heart transplantation at La Fe University and Polytechnic Hospital between October 2016 and April 2017 were considered eligible for inclusion. No additional clinical selection criteria were applied for study participation beyond those required for heart transplantation according to the institutional transplant program and current international guidelines. Exclusion criteria were death during the initial postoperative period and retransplantation. Samples were collected during follow-up visits of the recipients. The hospital follows an established rejection-monitoring protocol in which the first biopsy is performed 10–12 days after the transplant, the next one 10 days after the first biopsy, then every 6 weeks until the sixth month, and finally every 2 months until 1-year post-transplantation. In this period, 62 consecutive samples from 28 patients were included. Serum samples that could not be extracted from four patients (*n* = 5) were excluded, as well as samples from 12 patients lacking a histopathological diagnosis (*n* = 17) due to insufficient or poor-quality tissue samples for analysis. Conclusively, 40 consecutive samples with different rejection grades belonging to 24 patients were included in this study (Figure 1A), so, randomly, there are patients with more than one sample (Figure 1B). Blood samples were collected immediately before EMB for laboratory analysis. Once the samples were collected, they were processed, and the serum was separated through centrifugation (Eppendorf Model 5415R Centrifuge, Eppendorf Ibérica S.L.U., Madrid, Spain) at 1500× *g* for 15 min at 4 °C, aliquoted, and immediately stored at −80 °C until analysis.

Patients were maintained on a standard immunosuppressive regimen, and rejection episodes were assessed according to the International Society for Heart and Lung Transplantation consensus report [17]. The associated clinical data were collected at the time of each biopsy. For each sample, we recorded the age, sex, body mass, primary heart disease that was the cause of transplantation, time interval between transplantation and inclusion in the study (i.e., obtaining the blood sample), biochemical markers, echocardiographic parameters, and other relevant clinical characteristics at the time of each biopsy (Table 1). The researchers were blind to the group assignments and outcome assessments for all experiments.

### 2.2. RNA Sequencing Analysis

An RNA sequencing analysis was performed on 40 serum samples from patients diagnosed with biopsy-proven allograft rejection (ACR grade 1R, *n* = 16 and ACR grade ≥ 2R, *n* = 12) and from patients who did not experience allograft rejection (nonrejection, *n* = 12). This methodology has been described in extensive detail by Tarazon et al. [18]. Briefly, RNA was extracted using NucleoSpin miRNA Plasma from Macherey-Nagel (Düren, Germany) following the protocol and instructions provided by the manufacturer. cDNA libraries were then pooled and sequenced through two lanes of 100 bp paired-end sequencing using an Illumina HiSeq 2500 sequencer (Illumina, San Diego, CA, USA).

### 2.3. Statistical Analysis

For continuous variables, data were expressed as median and interquartile range, and as a percentage for discrete variables. Continuous clinical variables which had a non-normal distribution were analyzed using a linear mixed-effects model, while a generalized linear mixed-effects model was used for discrete clinical variables. The DESeq2 method (version 3.4) was used to perform an internal normalization of data [19]. The false discovery rate (FDR) method was used to adjust the original *p* value using the number of tests to prevent the identification of false positives in the differential expression data. An interpretation of this method was implemented in DESeq2, in which genes were ranked based on the *p*-value and multiplied by a correction rank value, similar to the original FDR method. In this exploratory study, the relative abundance of the mRNAs in the study groups was compared using a linear mixed-effects model. The study was primarily designed to collect independent observations. The additional observations were not systematically planned. The outcome variable, each of the fibrosis-related molecules examined in this study, was analyzed in its original form without transformation. The model included rejection grade as a fixed effect and patients as a random effect. To account for within-subject variability, a “no structure” covariance option was employed. This flexible strategy allows each variance and covariance between repeated measurements to be freely estimated, which was considered appropriate given that only a small number of cases involved repeated measurements within the same patient. The model employed the identity link function, which is the default for continuous outcomes in linear mixed models. The diagnostic capacity of fibrosis-related serum mRNA molecule-coding proteins for the presence of transplant rejection was evaluated. For this, receiver operating characteristic (ROC) curves were constructed to determine the sensitivity and specificity of the selected mRNA molecules. To calculate specificity and sensitivity, we took as a selection criteria an often-used cut-off point of FC ≥ 1.5. Molecules with significant discrimination capability (area under the curve [AUC] > 0.8) for detecting heart transplant rejection were selected. A *p* < 0.05 was considered to define a statistically significant difference. All statistical analyses were performed using the SPSS software version 20 for Windows (version 20.0; IBM SPSS Inc., Armonk, NY, USA), R (version R-4.3.1), and GraphPad Prism 8 (Dotmatics, Woburn, MA, USA).

## 3. Results

### 3.1. Clinical Characteristics of Patients

The study populations included in this transcriptomics study are presented in Table 1. All groups showed similar clinical characteristics with regard to variables such as age, sex, body mass, and comorbidities such as diabetes mellitus and immunosuppressive therapy. Only patients with ACR grade ≥ 2R displayed significant differences between specific clinical characteristics when we compared them with the nonrejection group. Patients with ACR grade ≥ 2R had the shortest time from the time of transplant until their incorporation into the study when compared with nonrejection patients (2.3 (0.4–6.8) vs. 9.2 (5.8–12) months, respectively; *p* < 0.01). In relation to the echo–Doppler study, we found, in patients with ACR grade ≥ 2R, higher values of the left ventricular end systolic diameter compared with the nonrejection group (32 (30–35) vs. 25 (23–29) mm, respectively; *p* < 0.05). Furthermore, these patients showed worse hemodynamic function compared with nonrejection patients since they had a higher right atrial pressure (7.5 (6.3–8.8) vs. 3.0 (2.0–5.5) mm Hg, respectively; *p* < 0.05), a higher systolic right ventricular pressure (44 (39–48) vs. 33 (29–38) mm Hg, respectively; *p* < 0.05) and a higher diastolic right ventricular pressure (7.0 (6.3–12) vs. 5.0 (2.5–5.5) mm Hg, respectively; *p* < 0.05). We also found, in patients with ACR grade ≥ 2R, higher values of neutrophils compared with the nonrejection group (6.3 (2.1–12) vs. 3.7 (2.5–4.4) thousands/mm^3^, respectively; *p* < 0.01) and higher values of leukocytes (11 (5.2–15) vs. 6.2 (5.3–6.7) thousands/mm^3^, respectively; *p* < 0.01). Moreover, we found an increase in N-terminal pro-B-type natriuretic peptide levels in patients with ACR grade ≥ 2R compared with those in the nonrejection group (1209 (736–2382) vs. 152 (113–467) pg/mL, respectively; *p* < 0.01).

### 3.2. Serum Relative Abundance of Genes Related to Fibrotic Process

Transcriptomics differences between the nonrejection and rejection groups were examined to determine whether cardiac allograft rejection was related to fibrosis. We identified alterations in the mRNAs of ECM and in those involved in the activation of fibroblast and myofibroblast, as well as in the TGF-β and WNT signaling pathways (Table 2).

**Figure 2:**
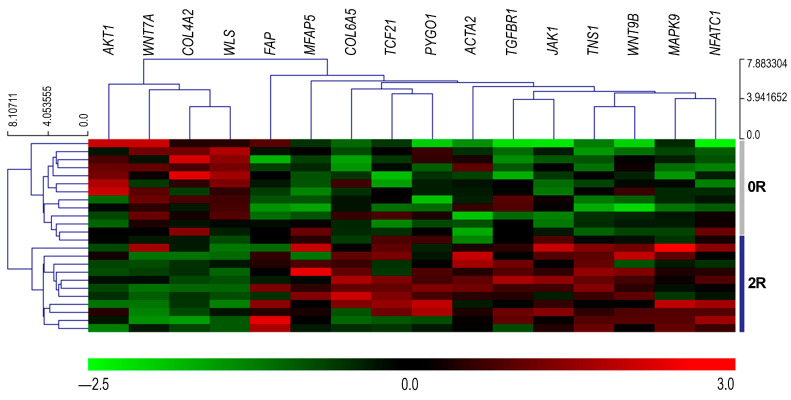
Serum relative abundance of genes related to the fibrotic process. Heat map summarizing mRNA relative abundance of genes related to the fibrotic process from the nonrejection (0R) and acute cellular rejection grade ≥ 2R groups. Each row represents a sample, and each column represents an altered mRNA. Colors depict the relative abundance of each molecule, with green being the lowest and red the highest.

In addition, altered genes were represented in a heat map and hierarchical clustering based on the fold change values using MeV software (version 4.9) [20]. Here, we observed two divergent mRNA profiles, indicating a clear distinction between nonrejection and ACR grade ≥ 2R groups (Figure 2). We have also observed that samples from the same patient are not closely linked in the dendrogram; hence, there is no evidence of dependence on the repeated measurements in this representation.

### 3.3. Serum mRNAs Encoding Components of the ECM

**Figure 3:**
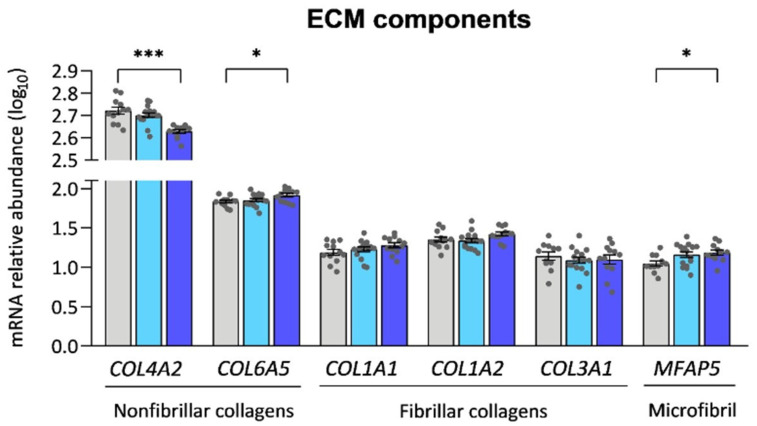
Circulating relative abundance of mRNAs encoding extracellular matrix proteins. Comparison of the serum mRNA relative abundance of the nonfibrillar collagens, the fibrillar collagens and extracellular matrix components between the nonrejection group and the groups representing various grades of acute rejection of heart allografts. Bars represent the mean values of mRNA relative abundance ± standard error of the mean value in log_10_ scale. The assigned colors denote the nonrejection group (gray), acute cellular rejection grade 1R group (light blue) and acute cellular rejection grade ≥ 2R group (dark blue). * *p* < 0.05; *** *p* < 0.001. *COL4A2*, collagen type IV alpha 2 chain; *COL6A5*, collagen type VI alpha 5 chain; *COL1A1*, collagen type I alpha 1 chain; *COL1A2*, collagen type I alpha 2 chain; *COL3A1*, collagen type III alpha 1 chain; *MFAP5*, microfibril-associated protein 5.

We identified 44 collagen genes (Table S1); however, only two of them (4.5%), compared to the nonrejection group, were differentially expressed in patients with ACR grade ≥ 2R, namely *COL4A2* and *COL6A5* (Figure 3). However, we did not find significant differences in the serum mRNA levels of fibrillar collagens I and III, which are the main markers of fibrosis (Figure 3). In addition, when we analyzed the relative abundance of different ECM components, we observed in patients with ACR grade ≥ 2R an increase in *MFAP5* mRNA relative abundance (Figure 3).

### 3.4. Serum mRNA Activators of Fibroblasts and Myofibroblasts 

**Figure 4:**
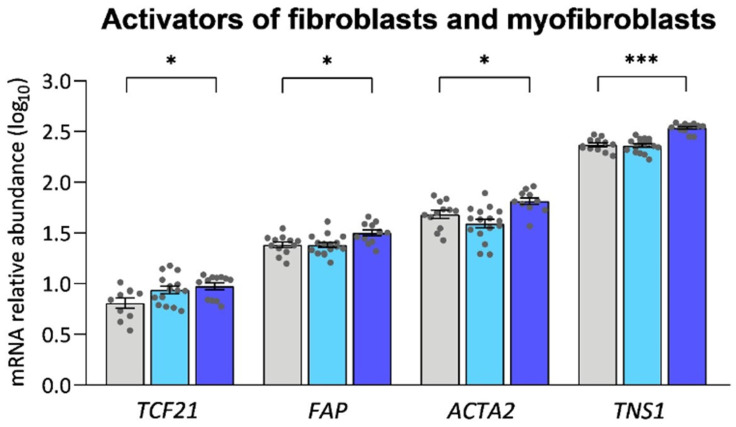
Circulating relative abundance of mRNAs encoding relevant activators of fibroblasts and myofibroblasts. Comparison of the serum mRNA relative abundance between the nonrejection group and the groups representing various grades of acute rejection of heart allografts. The assigned colors denote the nonrejection group (gray), acute cellular rejection grade 1R group (light blue) and acute cellular rejection grade ≥ 2R group (dark blue). Bars represent the mean values of mRNA relative abundance ± standard error of the mean value in log10 scale. * *p* < 0.05; *** *p* < 0.001. *TCF21*, transcription factor 21; *FAP*, fibroblast activation protein; *ACTA2*, alpha-smooth muscle actin; *TNS1*, tensin-1.

Since myocardial fibrosis is driven by the activation of fibroblasts, we wanted to evaluate whether serum mRNA abundances of the main markers of fibroblasts and myofibroblasts were altered. We observed significant differences in the relative abundance of several activators of fibroblasts and myofibroblasts when we compared the nonrejection group with patients with ACR grade ≥ 2R, such as *TCF21*, *FAP*, *ACTA2* and *TNS1* (Figure 4).

### 3.5. Serum mRNAs Encoding Proteins Involved in the TGF-β and WNT Signaling Pathways

**Figure 5:**
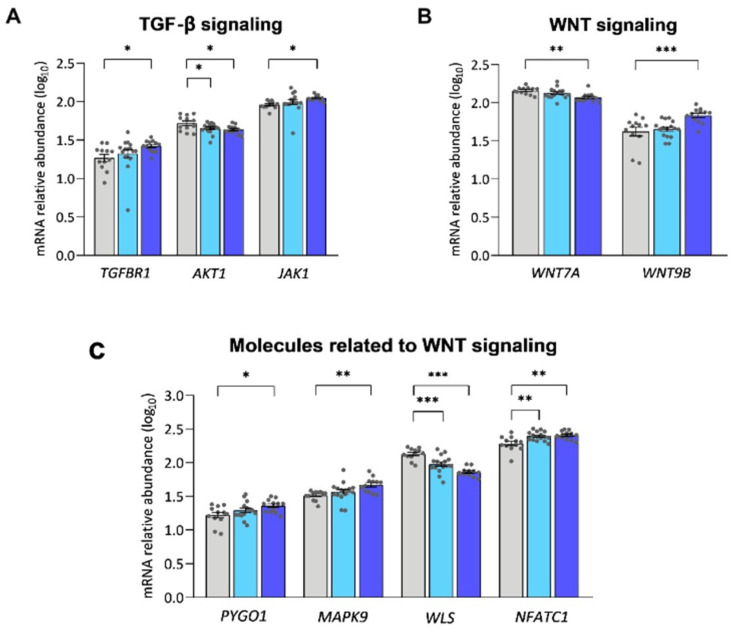
Circulating relative abundance of mRNAs encoding proteins involved in the TGF-β and WNT signaling pathways. Comparison of the serum mRNA relative abundance between the nonrejection group and the groups representing various grades of acute rejection of heart allografts of TGF-β signaling (**A**), WNT signaling (**B**) and proteins related to WNT signaling (**C**). Bars represent the mean values of mRNA relative abundance ± standard error of the mean value in log10 scale. The assigned colors denote the nonrejection group (gray), acute cellular rejection grade 1R group (light blue) and acute cellular rejection grade ≥ 2R group (dark blue). * *p* < 0.05; ** *p* < 0.01; *** *p* < 0.001. *TGFBR1*, transforming growth factor beta receptor 1; *AKT1*, RAC-alpha serine/threonine protein kinase 1; *JAK1*, Janus-Kinase 1; *WNT7A*, Wnt family member 7A; *WNT9B*, Wnt family member 9B; *PYGO1*, Pygopus family PHD finger 1; *MAPK9*, mitogen-activated protein kinase 9; *WLS*, Wntless; *NFATC1*, nuclear factor of activated T cells 1.

In light of our observation that several mRNAs related to the activation of fibrosis showed altered relative abundance in the serum of patients with ACR grade ≥ 2R, we probed whether the relative abundance of serum mRNAs of proteins involved in the major signaling pathways of the fibrotic process were also altered in these patients. First, we analyzed the TGF-β signaling pathway, which has a key role in the development of fibrosis. We found that, in the comparison between nonrejection and ACR grade ≥ 2R groups, there was an increase in the abundance of the TGF-β type 1 receptor (*TGFBR1*), which has a crucial role in the activation of canonical and noncanonical pathways of TGF-β signaling. In addition, we observed significant differences in the relative abundance of molecules that participate in noncanonical pathways such as *AKT1* and *JAK1*. *AKT1* relative abundance was also altered in patients with ACR grade 1R (Figure 5A).

We also analyzed the WNT signaling pathway because it is known to intervene in the pathological development of fibrosis through both canonical and noncanonical pathways. Therefore, we examined the relative abundance of circulating mRNAs encoding WNT proteins in patients with ACR grade ≥ 2R. We found that, in these patients, the abundance of WNT, which shows an affinity for both pathways, was altered. Specifically, *WNT7A* abundance was significantly lower in contrast to *WNT9B*, whose abundance increased in ACR grade ≥ 2R patients (Figure 5B).

Furthermore, we analyzed the relative abundance of serum mRNAs encoding proteins that actively participate in WNT signaling (Figure 5C). Compared with the nonrejection group, we found that patients with ACR grade ≥ 2R showed altered relative abundance in several molecules participating in the WNT pathway. We observed differences in the relative abundance of *PYGO1*, a mediator of WNT signaling, and *MAPK9*, which participates in the noncanonical pathway. Further, we observed differences in the relative abundance of *WLS*, a regulator of WNT protein secretion, and *NFATC1*, which is related to the noncanonical pathway. Moreover, *WLS* and *NFATC1* relative abundances were altered in patients with ACR grade 1R (Figure 5C).

### 3.6. Circulating Fibrosis-Related mRNAs Detect Acute Cellular Rejection

Considering our findings, we wanted to investigate the diagnostic capacity of ECM-related mRNAs and those of fibroblast and myofibroblast activators and TGF-β and WNT signaling pathways to detect cardiac allograft rejection. Therefore, ROC curves were obtained, and these data are summarized in Table 3, Figures S1 and S2, and the Supplementary Materials. Compared with the nonrejection group, we observed several molecules that presented an AUC > 0.80 to detect ACR grade ≥ 2R. Here, we also identified molecules that produced an AUC > 0.90 to differentiate patients with ACR grade ≥ 2R from those without rejection, specifically *TNS1* (AUC = 0.97, *p* < 0.001) and *WLS* (AUC = 0.99, *p* < 0.001) (Figure S1). Additionally, some molecules presented significant differences in AUC scores when we compared the group of patients without rejection to the group of patients with mild rejection (grade 1R), maintaining a remarkable and significant detection power. The molecules with a good capability to detect ACR grade 1R were *WLS* (AUC = 0.84, *p* < 0.01) and *NFATC1* (AUC = 0.83, *p* < 0.01) (Figure S2).

## 4. Discussion

Cardiac allograft rejection continues to represent a challenge in the heart transplantation field because immune injury contributes to graft failure [21] and cardiac graft vasculopathy [22]. Although the incidence of treated rejection and mortality after transplantation has continued to decrease due to maintenance immunosuppression, 12.6% of patients were treated for rejection between discharge and 1 year after transplantation [23], and an increased incidence was detected in the latest published report [24]. Given the invasive nature and technical limitations of EMBs, many recent studies have focused on the search for circulating biomarkers as a noninvasive alternative [25]. Myocardial fibrosis has been reported in previous studies using serial EMB of cardiac allografts. Gramley et al. observed extensive and progressive tissue remodeling in cardiac allografts, and myocardial fibrosis was central to this process [26]. In addition, Hughes et al. employed magnetic resonance imaging to reveal a close association between the prevalence of myocardial fibrosis and the severity of cardiac allograft vasculopathy and with a higher incidence of all-cause death or major adverse cardiac events [11]. Consistent with these findings, previous cardiovascular magnetic resonance studies have shown that heart transplant recipients exhibit impaired cardiac mechanics compared with controls, accompanied by increased myocardial fibrosis, with no significant differences between donation after circulatory death and donation after brain death transplants, despite the longer warm ischemic time associated with donation after circulatory death [27]. However, whether ACR is related to fibrotic processes in patients with heart transplant remains unclear. This current exploratory study used RNA-seq analysis of serum samples to arrive at new insights into the activation of the fibrotic process in patients with cardiac transplant who were diagnosed with ACR.

Regarding myocardial fibrosis, our results showed that 4.5% of all collagens were significantly altered in patients with ACR grade ≥ 2R compared with nonrejection patients. Among these, we did not find significant differences in the serum mRNA levels of fibrillar collagens I and III, whose secretion is a hallmark of cardiac fibrosis [28]. The increase in relative abundance of several nonfibrillar collagens has also been associated with cardiac fibrosis [14]. In this study, we observed *COL6A5* and *COL4A2* to be dysregulated in patients with ACR grade ≥ 2R. It has been previously reported that collagen VI promotes myofibroblast conversion and enhances fibrosis in experimental myocardial infarction (MI) [14]. In a study of patients undergoing renal transplant surgery, Stribos et al. observed increased collagen type VI expression in fibrotic lesions. In addition, circulating PRO-C6 levels in plasma reflect the stage of chronic kidney disease, and levels significantly increase with disease severity [29]. This issue requires further investigation given that there are no previous studies on the abundance patterns of nonfibrillar collagens in cardiac allograft rejection.

Considering that the activation of cardiac fibroblasts is one of the main cardiac fibrosis-related signaling pathways, we focused on its analysis in patients with ACR grade ≥ 2R. First, we found that several activated fibroblast markers increased their relative abundance, which may indicate that cardiac fibroblasts had become activated and acquired greater relevance in cardiac pathological conditions as the main producers of type I and III collagens [30]. Among them, we highlighted the detected higher relative abundance of *FAP*, since we have recently provided new insights into the central role this protein plays in the process of cardiac fibrosis, particularly in the activation of cardiac fibroblasts in patients with HF [31]. Interestingly, in response to injury, fibroblasts differentiate into highly specialized synthetic and contractile myofibroblasts, which have a great capacity to produce ECM proteins and increase fibrillar collagen accumulation, resulting in myocardial fibrosis [32]. In this study, we found that, in patients with ACR grade ≥ 2R, compared with nonrejection patients, there was a higher relative abundance of markers of activated myofibroblasts, such as *ACTA2* and *TNS1*. In addition to synthesizing fibrillar collagens, activated cardiac fibroblasts are responsible for the deposition of new ECM after injury and during the remodeling phases of wound healing [33]. We observed significant differences in the mRNA relative abundance of ECM components related to fibrosis, such as *MFAP5* which may promote scar formation and fibrosis [33]. These findings are manifest signs of activation of the fibrotic process in patients with rejection, since we observed activation of fibroblast and myofibroblast markers that could initiate the production of fibrillar collagens and ECM proteins. This topic requires further investigation, given the exploratory nature of the study and that there is no previous evidence of any relationship or regulation between confounding factors related to rejection status, such as response time or immunosuppressive burden and fibrosis activation, in patients diagnosed with ACR.

In line with these results, we analyzed the different repair signaling pathways involved to ensure that the fibrotic process was being activated. First, TGF-β higher relative abundance has been previously linked with chronic rejection and may negatively impact graft survival through chemotactic and profibrotic effects [7]. It has also been observed that TGF-β stimulation potently induces myofibroblast conversion and increases ECM protein synthesis by activated fibroblasts [14]. In a murine model of chronic transplant rejection, Brunner et al. found that TGF-β1 is produced by macrophages after stimulation by IL-13 via IL-13Ra2. They observed that blockage of the IL-13/IL-13Ra2 pathway with an siRNA could prevent cardiac allograft fibrosis, marking IL-13-Ra2 as an exploitable therapeutic target for preventing allograft fibrosis [34]. TGF-β1 and its two receptors, TGFβR1 and TGFβR2, play key roles in epithelial–mesenchymal transition and fibrogenesis [35]. Tang et al. identified JAK1 as a TGFR1-interacting protein and showed that JAK1 activates STAT3 via both SMAD-independent and -dependent mechanisms due to the TGF-induced fibrotic response in hepatic cells [36]. When we analyzed TGF-β signaling, we found alterations in *TGFBR1*, *JAK1* and *AKT1* mRNA relative abundance in patients with ACR grade ≥ 2R compared with nonrejection patients. Both JAK/STAT and PI3K/Akt signaling play key roles in the occurrence and development of myocardial fibrosis, which is directly or indirectly related to the modulation of cardiac fibroblasts and/or ECM [37,38]. These results indicate an activation of the TGF-β signaling pathway, which has an important role in the activation of the fibrotic process.

On the other hand, we also found significant differences in the mRNA relative abundance of molecules that participate in the WNT signaling pathway, such as *WNT7A* and *WNT9B*, which show a preference for noncanonical and canonical pathways [39]. Ye et al. have previously suggested an association between canonical WNT/β-catenin signaling and epicardial fibrosis in failed pediatric heart allografts [40]. Additionally, we observed ACR grade-dependent differences in *WLS* mRNA relative abundance, which is involved in the secretion of WNT proteins [41] and whose deletion promotes cardiac fibroblast activation post-MI [42]. Despite the lack of knowledge about the role of WNT signaling in the physiopathology of ACR and the development of fibrosis in this pathology, our results suggest that this pathway could also play an important role in the development of fibrosis associated with cardiac rejection. Recent reports have highlighted the extensive crosstalk between the TGF-β and WNT pathways, which could be responsible for the transcription of profibrotic genes [43]. Blyszczuk et al. proposed that the targeting of WNT proteins or the WNT pathway may represent an attractive alternative for anti-TGF-β treatment by offering a broader range of available pharmacological compounds with well-defined function. They observed that the inhibition of WNT signaling improved cardiac function, indicating that pharmacological targeting of extracellular WNT proteins or the canonical WNT pathway may represent a promising therapeutic option. Antifibrotic therapy in patients with inflammatory cardiomyopathy can potentially prevent disease progression [44]. Further studies are necessary to delve into the relationship between TGF-β and WNT pathways in patients with ACR. Therefore, our analysis of the serum of patients with varying grades of acute cardiac allograft rejection and immunosuppressive load revealed an activation of the fibrotic process, given that we discovered that pertinent genes, like *FAP* or *ACTA2*, which are associated with the activation of fibroblasts and myofibroblasts, as well as the major profibrotic pathways, namely TGF-β and WNT signaling, exhibit differential relative abundance. Interestingly, patients with clinically relevant rejection showed most of these alterations in relative abundance compared with nonrejection patients. As mentioned above, it is crucial to provide a noninvasive alternative to EMB to achieve an optimal approach for diagnosing cardiac allograft rejection, especially in the early phase [45]. In the present exploratory study, among the altered molecules related to the fibrotic process found in patients with ACR, *TNS1* and *WLS* had the best diagnostic capabilities for identifying heart transplant rejection grade ≥ 2R. This issue requires further investigation and external validation before their potential implementation in routine clinical surveillance. Collectively, these findings highlight the relevance of these molecules in the context of cardiac allograft rejection and profibrotic signaling and their potential utility as exploratory biomarkers for the detection of ACR in heart transplant recipients.

We acknowledge several limitations of this study, and the results must be interpreted within this context. This investigation involved only a single center, focused on cellular rejection, and did not specifically evaluate antibody-mediated rejection, and, given the sample size, extensive exploration of the effect of clinical variables on mRNA relative abundance is not feasible. Given our limited cohort, future independent external studies are needed to examine our findings using large patient populations, including confounders such as immunosuppressive load or the early post-transplant timing of rejection events. However, we present our findings as the first step in supporting future research focused on the inhibition of fibrotic process activation in patients with ACR. In addition, we provide new insights into the search for biomarkers related to fibrosis process activation, not only with the objective of detecting and targeting fibrosis, but also as early biomarkers of cardiac rejection in patients with cardiac transplant. Hence, future validation studies with larger cohorts and an in-depth analysis of the pathophysiological implications of the fibrotic process are needed.

## 5. Conclusions

We found alterations in the relative abundance of circulating activators of fibroblasts and myofibroblasts, such as *FAP* or *ACTA2*, as well as in major profibrotic pathways, including TGF-β and WNT signaling, especially in clinically relevant cardiac rejection. These findings may contribute to improving the surveillance of patients with cardiac transplant and provide new therapeutic strategies for targeting fibrosis process activation. Future studies in larger cohorts are needed to better understand the potential impact of confounding variables.

## Figures and Tables

**Table 1:** Patient characteristics at the time of blood sample extraction.

	Nonrejection (*n* = 12)	Rejection (*n* = 28)		*p* Value ^a^
		Grade 1R (*n* = 16)	Grade ≥ 2R (*n* = 12)	
Age, years	55 (35–60)	49 (43–60)	42 (29–57)	0.573
Male sex (%)	75	94	75	0.939
Indication for cardiac transplantation				
Ischemic cardiomyopathy (%)	25	50	25	0.999
Idiopathic dilated cardiomyopathy (%)	42	31	58	0.839
Other (%)	33	19	17	0.838
Time between transplantation and study enrollment (months)	9.2 (5.8–12)	5.4 (3.8–9.7)	2.3 (0.4–6.8)	0.003
Body mass index (kg/m^2^)	25 (24–26)	25 (23–27)	25 (22–26)	0.813
Hypertension (%)	58	31	42	0.592
Diabetes mellitus (%)	58	63	50	0.995
Dyslipemia (%)	42	56	25	0.844
Echo–Doppler study				
Ejection fraction (%)	77 (70–77)	63 (60–68)	70	0.759
LV end systolic diameter (mm)	25 (23–29)	28 (24–32)	32 (30–35)	0.018
LV end diastolic diameter (mm)	42 (39–44)	43 (43–47)	46 (44–47)	0.262
Hemodynamic parameters				
Mean right atrial pressure (mm Hg)	3.0 (2.0–5.5)	6.0 (3.5–7.0)	7.5 (6.3–8.8)	0.031
Systolic right ventricular pressure (mm Hg)	33 (29–38)	38 (32–42)	44 (39–48)	0.021
Diastolic right ventricular pressure (mm Hg)	5.0 (2.5–5.5)	5.0 (3.0–7.8)	7.0 (6.3–12)	0.027
Immunosuppressive therapy				
Tacrolimus (%)	100	100	100	1.000
Mycophenolic acid (%)	100	100	100	1.000
Steroids (%)	100	100	100	1.000
Induction therapy				
Basiliximab (%)	100	100	100	1.000
Neutrophils (thousands/mm^3^)	3.7 (2.5–4.4)	3.5 (2.7–5.2)	6.3 (2.1–12)	0.001
Leukocytes (thousands/mm^3^)	6.2 (5.3–6.7)	6.2 (5.1–7.7)	11 (5.2–15)	0.001
Lymphocytes (thousands/mm^3^)	1.6 (1.4–1.8)	1.8 (1.3–2.0)	2.1 (1.3–2.6)	0.126
Hemoglobin (g/dL)	11 (10–14)	13 (11–14)	12 (10–14)	0.672
Hematocrit (%)	34 (33–43)	40 (35–42)	36 (34–42)	0.684
NT-proBNP (pg/mL)	152 (113–467)	280 (122–572)	1209 (736–2382)	0.002
Troponin T (ng/L)	19 (11–66)	15 (10–21)	25 (13–40)	0.354

^a^ Nonrejection vs. acute cellular rejection grade ≥ 2R; data are presented as median (interquartile ranges); LV: left ventricle; NT-proBNP: N-terminal fragment of B-type natriuretic peptide.

**Table 2:** Alterations in the relative abundance of mRNAs encoding proteins related to fibrosis process in nonrejection, grade 1R and grade 2R groups.

Gene Name	Nonrejection	Grade 1R	Grade 2R	*p* Value ^a^	*p* Value ^b^
Components of the extracellular matrix					
*COL4A2*	525 (468–574)	501 (485–524)	433 (409–441)	ns	<0.001
*COL6A5*	69 (63–72)	71 (63–84)	88 (68–94)	ns	0.012
*MFAP5*	10 (10–14)	16 (11–18)	15 (13–18)	ns	0.033
Activators of fibroblasts and myofibroblasts					
*TCF21*	7.3 (4.6–8.5)	8.6 (6.1–11)	11 (6.8–12)	ns	0.033
*FAP*	25 (21–28)	24 (20–28)	32 (27–39)	ns	0.009
*ACTA2*	51 (38–62)	42 (32–51)	65 (57–78)	ns	0.030
*TNS1*	232 (212–260)	234 (201–264)	356 (327–373)	ns	<0.001
Proteins involved in the *TGF-β* signaling pathway					
*TGFBR1*	20 (15–24)	23 (19–27)	27 (23–30)	ns	0.024
*AKT1*	53 (42–66)	47 (42–50)	43 (41–47)	0.048	0.027
*JAK1*	92 (86–100)	98 (93–113)	111 (106–120)	ns	0.045
Proteins involved in the WNT signaling pathway					
*WNT7A*	147 (132–155)	131 (122–144)	114 (107–125)	ns	0.003
*WNT9B*	50 (37–55)	46 (37–56)	73 (58–79)	ns	<0.001
*PYGO1*	18 (15–22)	20 (17–23)	25 (19–27)	ns	0.030
*MAPK9*	35 (29–37)	38 (33–42)	48 (37–53)	ns	0.003
*WLS*	136 (124–158)	93 (85–110)	72 (66–77)	<0.001	<0.001
*NFATC1*	195 (168–234)	245 (220–288)	255 (226–292)	0.003	0.003

^a^ Comparison between nonrejection and acute cellular rejection grade 1R; ^b^ comparison between nonrejection and acute cellular rejection grade ≥ 2R. Data are presented as median (interquartile ranges). *COL4A2*: collagen type IV alpha 2 chain; *COL6A5*: collagen type VI alpha 5 chain; *MFAP5*: microfibril-associated protein 5; *TCF21*: transcription factor 21; *FAP*: fibroblast activation protein; *ACTA2*: alpha-smooth muscle actin; *TNS1*: tensin-1; *TGFBR1*: transforming growth factor beta receptor 1; *AKT1*: RAC-alpha serine/threonine protein kinase 1; *JAK1*: Janus-Kinase 1; *WNT7A*: Wnt family member *7A*; *WNT9B*: Wnt family member 9B; *PYGO1*: Pygopus family PHD finger 1; *MAPK9*: mitogen-activated protein kinase 9; *WLS*: Wntless; *NFATC1*: nuclear factor of activated T cells 1.

**Table 3:** Receiver operating characteristic (ROC) curve of circulating altered genes related to fibrosis process for detecting heart transplant rejection (grade ≥ 2R). Sensitivities and specificities for the diagnosis of cardiac rejection (cut-off point FC ≥ 1.5).

Gene Name	AUC	95% CI	SS	95% CI	SP	95% CI
Components of the extracellular matrix						
*MFAP5*	0.82 *	0.636–1.000	25	(0.055–0.572)	91	(0.587–0.997)
Activators of fibroblasts and myofibroblasts						
*TNS1*	0.97 ***	0.918–1.000	50	(0.211–0.789)	100	(0.735–1.000)
*TCF21*	0.81 *	0.621–0.996	67	(0.349–0.901)	100	(0.735–1.000)
*FAP*	0.81 *	0.628–0.983	25	(0.055–0.572)	100	(0.735–1.000)
*ACTA2*	0.80 *	0.620–0.986	33	(0.099–0.651)	100	(0.735–1.000)
Proteins involved in the TGF-β signaling pathway						
*TGFBR1*	0.81 *	0.639–0.986	33	(0.099–0.651)	100	(0.735–1.000)
Proteins involved in the WNT signaling pathway						
*WLS*	0.99 ***	0.950–1.000	33	(0.099–0.651)	100	(0.735–1.000)
*WNT9B*	0.88 **	0.745–1.000	58	(0.277–0.848)	92	(0.615–0.998)
*MAPK9*	0.88 **	0.741–1.000	42	(0.152–0.723)	100	(0.735–1.000)
*NFATC1*	0.85 **	0.700–1.000	17	(0.021–0.484)	100	(0.735–1.000)
*PYGO1*	0.82 *	0.650–0.989	25	(0.055–0.572)	100	(0.735–1.000)

Comparison between nonrejection group and grade ≥ 2R acute cellular rejection: * *p* < 0.05; ** *p* < 0.01; *** *p* < 0.001. AUC: area under the curve; CI: confidence interval; SS: sensitivity; SP: specificity. *MFAP5*: microfibril-associated protein 5; *TNS1*: tensin-1; *TCF21*: transcription factor 21; *FAP*: fibroblast activation protein; *ACTA2*: alpha-smooth muscle actin; *TGFBR1*: transforming growth factor beta receptor 1; *WLS*: Wntless; *WNT9B*: Wnt family member 9B; *MAPK9*: mitogen-activated protein kinase 9; *NFATC1*: nuclear factor of activated T cells 1; *PYGO1*: Pygopus family PHD finger 1.

## Data Availability

The RNA-seq data discussed in this publication have been deposited in NCBI’s Gene Expression Omnibus [47] and are accessible through GEO Series Accession Number GSE315025 (https://www.ncbi.nlm.nih.gov/geo/query/acc.cgi?acc=GSE315025, accessed on 15 March 2023).

## Supplementary Materials

The following supporting information can be downloaded at: https://www.mdpi.com/article/10.3390/biomedicines14061371/s1, Figure S1: Receiver operating characteristic (ROC) curve of coding serum mRNA of altered genes related to fibrosis process for the detection of cardiac allograft rejection (ACR grade ≥ 2R); Figure S2: Receiver operating characteristic (ROC) curve of coding serum mRNA of altered genes related to fibrosis process for the detection of cardiac allograft rejection (ACR grade 1R); Table S1: Detected serum collagens in ACR patients.

## Abbreviations

The following abbreviations are used in this manuscript:

ACR	Acute Cellular Rejection
AUC	Area Under the Curve
ECM	Extracellular Matrix
EMB	EndoMyocardial Biopsy
FDR	False Discovery Rate
MI	Myocardial Infarction
ROC	Receiver Operating Characteristic
TGF-β	Transforming Growth Factor Beta
