# Supplementary material for: Fibrosis Process Activation in Patients with Acute Cardiac Rejection: A Novel Noninvasive Diagnostic Approach
Source: Biomedicines. 2026 Jun 18;14(6):1371. doi: 10.3390/biomedicines14061371 (PMC13297594; doi:10.3390/biomedicines14061371)
Supplement: Supplementary file 1 [file biomedicines-14-01371-s001.zip › biomedicines-4302891-supplementary.pdf]

SUPPLEMENTARY MATERIAL

Fibrosis Process Activation in Patients with Acute Cardiac Rejection: A Novel Noninvasive Diagnostic Approach

Marta Delgado-Arija [1,2], Lorena Pérez-Carrillo [1,2], Irene González-Torrent [1], Patricia Genovés [2,3], Isaac Gimé-nez-Escamilla [1,2], Carlota Benedicto [1], Luis Martínez-Dolz [1,2,4], Manuel Portolés [1,2], Estefanía Tarazón [1,2,*,†] and Esther Roselló-Lletí [1,2,*,†]

[1] Clinical and Translational Research in Cardiology Unit, Health Research Institute Hospital La Fe (IIS La Fe), Avd. Fernando Abril Martorell 106, 46026 Valencia, Spain; marta_delgado@externos.iislafe.es (M.D.-A.); lorena_perezc@iislafe.es (L.P.-C.); irene_gonzalez@iislafe.es (I.G.-T.); isaacgimene-zuv@gmail.com (I.G.-E.); carlota_benedicto@iislafe.es (C.B.); martinez_luidol@gva.es (L.M.-D.); drmanuelportoles@gmail.com (M.P.)

[2] Center for Biomedical Research Network on Cardiovascular Diseases (CIBERCV), Avd. Monforte de Lemos 3-5, 28029 Madrid, Spain; pagenomar@gmail.com

[3] Department of Biomedical Sciences, CEU Cardenal Herrera University, Street Santiago Ramón y Cajal 20, 46115 Valencia, Spain

[4] Heart Failure and Transplantation Unit, Cardiology Department, University and Polytechnic La Fe Hospital, Avd. Fernando Abril Martorell 106, 46026 Valencia, Spain

* Correspondence: estefania_tarazon@iislafe.es (E.T.); esther_rosello@iislafe.es (E.R.-L.); Tel.: +34-96-124-66-44 (E.T. & E.R.-L.)

† These authors contributed equally to this work.

TABLE OF CONTENTS

Supplementary Figures………………………………………..……………………..3
Figure S1. Receiver operating characteristic (ROC) curve of coding serum mRNA of altered genes related to fibrosis process for the detection of cardiac allograft rejection (ACR grade ≥2R).
Figure S2. Receiver operating characteristic (ROC) curve of coding serum mRNA of altered genes related to fibrosis process for the detection of cardiac allograft rejection (ACR grade 1R).
Supplementary Tables……………………………………………………………......5
Table S1: Detected serum collagens in ACR patients

SUPPLEMENTARY FIGURES

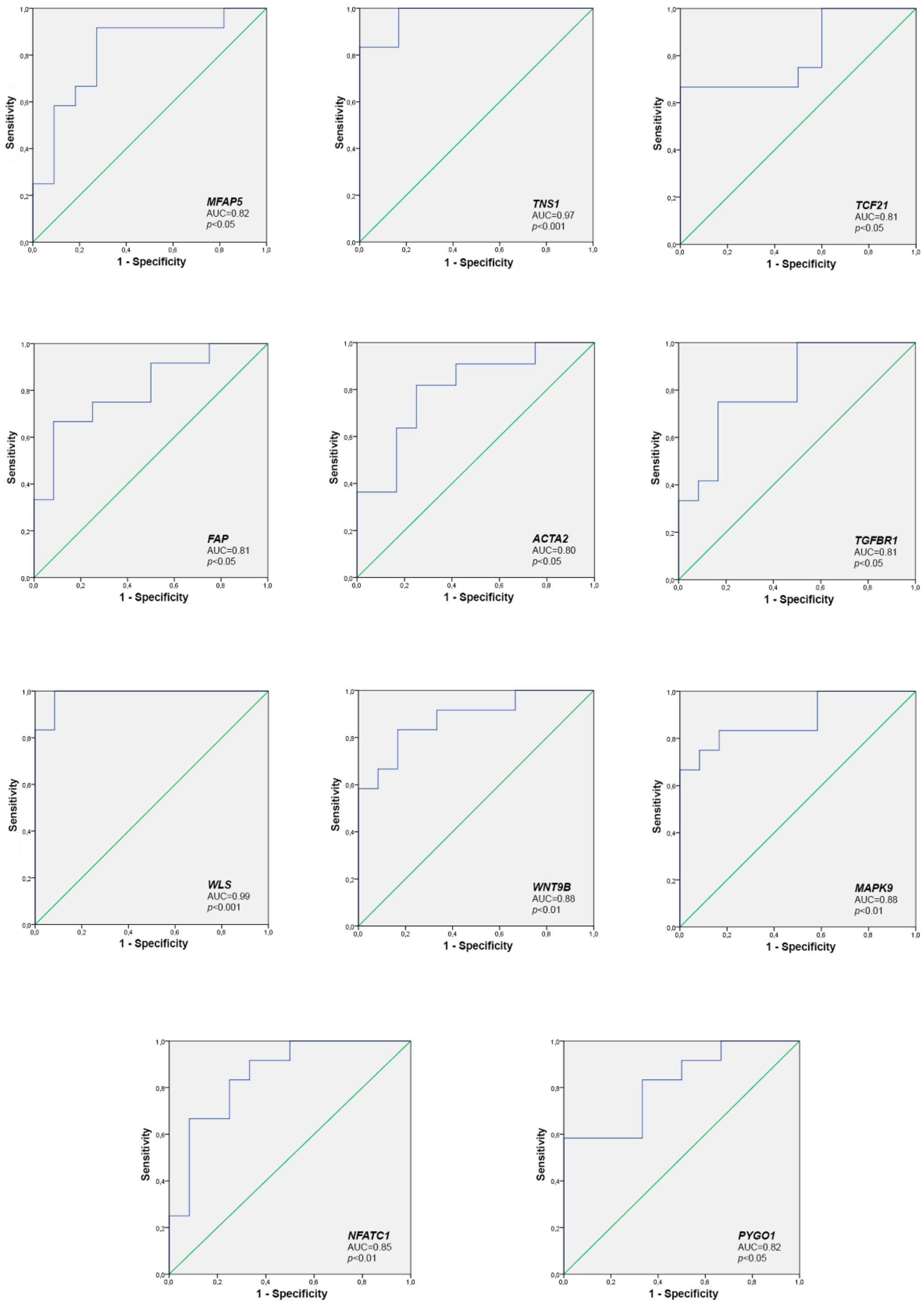

Figure S1. Receiver operating characteristic (ROC) curve of coding serum mRNA of altered genes related to fibrosis process for the detection of cardiac allograft rejection (ACR grade ≥2R). AUC: Area under the curve. *MFAP5*-Microfibril associated protein 5; *TNS1*-Tensin-1; *TCF21*-Transcription factor 21; *FAP*-Fibroblast activation protein; *ACTA2*-Alpha-smooth muscle actin; *TGFBR1*-Transforming growth factor beta receptor 1; *WLS*-Wntless; *WNT9B*-Wnt family member 9B; *MAPK9*-Mitogen-activated protein kinase 9; *NFATC1*-Nuclear factor of activated T cells 1; *PYGO1*-Pygopus family PHD finger 1.

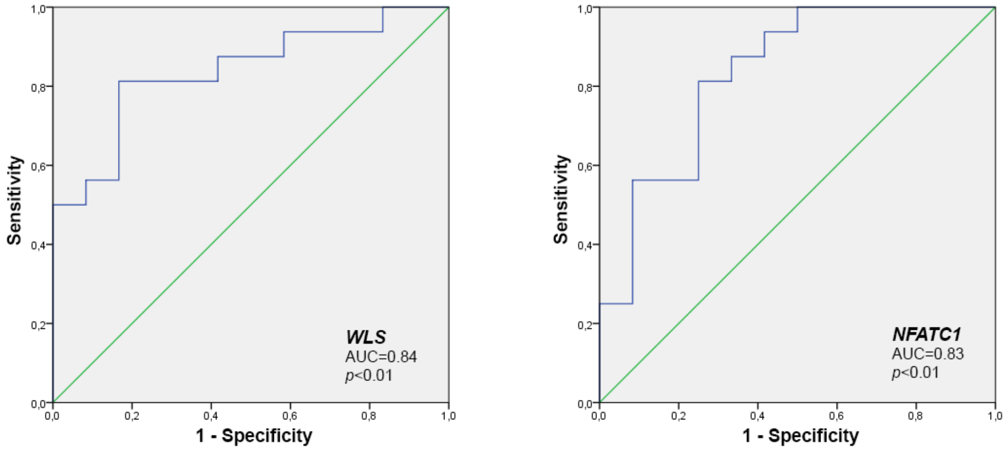

Figure S2. Receiver operating characteristic (ROC) curve of coding serum mRNA of altered genes related to fibrosis process for the detection of cardiac allograft rejection (ACR grade 1R). AUC: Area under the curve. *WLS*-Wntless; *NFATC1*-Nuclear factor of activated T cells 1.

SUPPLEMENTARY TABLES

Table S1: Detected serum collagens in ACR patients

	Ensembl ID	Gene name	Protein name (UniProtKB)
1	ENSG00000108821	*COL1A1*	Collagen alpha-1 (I) chain
2	ENSG00000164692	*COL1A2*	Collagen alpha-2 (I) chain
3	ENSG00000139219	*COL2A1*	Collagen alpha-1 (II) chain
4	ENSG00000168542	*COL3A1*	Collagen alpha-1 (III) chain
5	ENSG00000187498	*COL4A1*	Collagen alpha-1 (IV) chain
6	**ENSG00000134871**	***COL4A2***	**Collagen alpha-2 (IV) chain**
7	ENSG00000169031	*COL4A3*	Collagen alpha-3 (IV) chain
8	ENSG00000081052	*COL4A4*	Collagen alpha-4 (IV) chain
9	ENSG00000188153	*COL4A5*	Collagen alpha-5 (IV) chain
10	ENSG00000197565	*COL4A6*	Collagen alpha-6 (IV) chain
11	ENSG00000130635	*COL5A1*	Collagen alpha-1 (V) chain
12	ENSG00000204262	*COL5A2*	Collagen alpha-2 (V) chain
13	ENSG00000080573	*COL5A3*	Collagen alpha-3 (V) chain
14	ENSG00000142156	*COL6A1*	Collagen alpha-1 (VI) chain
15	ENSG00000142173	*COL6A2*	Collagen alpha-2 (VI) chain
16	ENSG00000163359	*COL6A3*	Collagen alpha-3 (VI) chain
17	**ENSG00000172752**	***COL6A5***	**Collagen alpha-5 (VI) chain**
18	ENSG00000206384	*COL6A6*	Collagen alpha-6 (VI) chain
19	ENSG00000114270	*COL7A1*	Collagen alpha-1 (VII) chain
20	ENSG00000144810	*COL8A1*	Collagen alpha-1 (VIII) chain
21	ENSG00000171812	*COL8A2*	Collagen alpha-2 (VIII) chain
22	ENSG00000112280	*COL9A1*	Collagen alpha-1 (IX) chain
23	ENSG00000049089	*COL9A2*	Collagen alpha-2 (IX) chain
24	ENSG00000092758	*COL9A3*	Collagen alpha-3 (IX) chain
25	ENSG00000123500	*COL10A1*	Collagen alpha-1 (X) chain
26	ENSG00000060718	*COL11A1*	Collagen alpha-1 (XI) chain
27	ENSG00000204248	*COL11A2*	Collagen alpha-2 (XI) chain
28	ENSG00000111799	*COL12A1*	Collagen alpha-1 (XII) chain
29	ENSG00000197467	*COL13A1*	Collagen alpha-1 (XIII) chain
30	ENSG00000187955	*COL14A1*	Collagen alpha-1 (XIV) chain
31	ENSG00000204291	*COL15A1*	Collagen alpha-1 (XV) chain
32	ENSG00000084636	*COL16A1*	Collagen alpha-1 (XVI) chain
33	ENSG00000065618	*COL17A1*	Collagen alpha-1 (XVII) chain
34	ENSG00000182871	*COL18A1*	Collagen alpha-1 (XVIII) chain
35	ENSG00000082293	*COL19A1*	Collagen alpha-1 (XIX) chain
36	ENSG00000101203	*COL20A1*	Collagen alpha-1 (XX) chain
37	ENSG00000124749	*COL21A1*	Collagen alpha-1 (XXI) chain
38	ENSG00000169436	*COL22A1*	Collagen alpha-1 (XXII) chain
39	ENSG00000050767	*COL23A1*	Collagen alpha-1 (XXIII) chain
40	ENSG00000171502	*COL24A1*	Collagen alpha-1 (XXIV) chain
41	ENSG00000188517	*COL25A1*	Collagen alpha-1 (XXV) chain

42	ENSG00000160963	*COL26A1*	Collagen alpha-1 (XXVI) chain
43	ENSG00000196739	*COL27A1*	Collagen alpha-1 (XXVII) chain
44	ENSG00000215018	*COL28A1*	Collagen alpha-1 (XXVIII) chain

Serum collagens highlighted in bold have altered expression in patients with ACR compared to nonrejection group.
ACR, acute cellular rejection
